# Term amniotic membrane is a high throughput source for multipotent mesenchymal stem cells with the ability to differentiate into endothelial cells in vitro

**DOI:** 10.1186/1471-213X-7-11

**Published:** 2007-02-21

**Authors:** Francesco Alviano, Valentina Fossati, Cosetta Marchionni, Mario Arpinati, Laura Bonsi, Michele Franchina, Giacomo Lanzoni, Silvia Cantoni, Claudia Cavallini, Francesca Bianchi, Pier Luigi Tazzari, Gianandrea Pasquinelli, Laura Foroni, Carlo Ventura, Alberto Grossi, Gian Paolo Bagnara

**Affiliations:** 1 Department of Histology, Embryology and Applied Biology, University of Bologna, Italy; 2 Institute of Hematology and Medical Oncology "Seragnoli", University of Bologna, Italy; 3 Department of Obstetrics and Gynecology, University of Bologna, Italy; 4 Laboratory of Molecular Biology and Stem Cell Engineering, National Institute of Biostructures and Biosystems at the Institute of Cardiology, University of Bologna, Italy; 5 Institute of Nephrology, Dialysis and Renal Transplantation, S. Orsola University Hospital, University of Bologna, Italy; 6 Department of Experimental Pathology, University of Bologna, Italy; 7 Department of Anesthesiological and Surgical Sciences, University of Bologna, Italy; 8 Stem Cell Research Center, University of Bologna, Italy; 9 Laboratory of Experimental Hematology, National Institute of Biostructures and Biosystems at the Department of Histology, Embryology and Applied Biology, University of Bologna, Italy; 10 Cardiovascular Tissue Bank, Department of Hematology, Oncology and Laboratory Medicine, S. Orsola University Hospital, Bologna, Italy

## Abstract

**Background:**

Term Amniotic membrane (AM) is a very attractive source of Mesenchymal Stem Cells (MSCs) due to the fact that this fetal tissue is usually discarded without ethical conflicts, leading to high efficiency in MSC recovery with no intrusive procedures. Here we confirmed that term AM, as previously reported in the literature, is an abundant source of hMSCs; in particular we further investigated the AM differentiation potential by assessing whether these cells may also be committed to the angiogenic fate. In agreement with the recommendation of the International Society for Cellular Therapy, the mesenchymal cells herein investigated were named Amniotic Membrane-human Mesenchymal Stromal Cells (AM-hMSC).

**Results:**

The recovery of hMSCs and their *in vitro *expansion potential were greater in amniotic membrane than in bone marrow stroma. At flow cytometry analysis AM-hMSCs showed an immunophenotypical profile, i.e., positive for CD105, CD73, CD29, CD44, CD166 and negative for CD14, CD34, CD45, consistent with that reported for bone marrow-derived MSCs. In addition, amniotic membrane-isolated cells underwent *in vitro *osteogenic (von Kossa stain), adipogenic (Oil Red-O stain), chondrogenic (collagen type II immunohistochemichal detection) and myogenic (RT-PCR MyoD and Myogenin expression as well as desmin immunohistochemical detection) differentiation. In angiogenic experiments, a spontaneous differentiation into endothelial cells was detected by *in vitro *matrigel assay and this behaviour has been enhanced through Vascular Endothelial Growth Factor (VEGF) induction. According to these findings, VEGF receptor 1 and 2 (FLT-1 and KDR) were basally expressed in AM-hMSCs and the expression of endothelial-specific markers like FLT-1 KDR, ICAM-1 increased after exposure to VEGF together with the occurrence of CD34 and von Willebrand Factor positive cells.

**Conclusion:**

The current study suggests that AM-hMSCs may emerge as a remarkable tool for the cell therapy of multiple diseased tissues. AM-hMSCs may potentially assist both bone and cartilage repair, nevertheless, due to their angiogenic potential, they may also pave the way for novel approaches in the development of tissue-engineered vascular grafts which are useful when vascularization of ischemic tissues is required.

## Background

MSCs are cells with high *in vitro *expansion potential and self renewal capacity which were first isolated from bone marrow [1-3]. Beside their ability to differentiate into multiple mesoderm-type lineages, e.g. osteoblasts, chondrocytes and adipocytes, bone marrow derived-MSCs are also able to differentiate into endothelial cells *in vitro *[4]; this opens new possibilities for promoting angiogenesis through cell-based therapeutic strategies.

Occlusive vascular disease is the most important cause of death and morbidity in industrialized countries. Treatment of end-stage disease, such as myocardium infarction, is usually accomplished by angioplasty, surgery, bypasses and other palliative interventions. However, patients with occlusive vascular disease develop a prominent collateral vascular network below the occlusive site through spontaneous arteriogenesis and angiogenesis to which contribute bone marrow derived-mesenchymal stem cells (MSCs) [5].

However, considering the invasive procedure related to this source, there is an increasing interest in investigating the presence of mesenchymal stem cells with angiogenic potentiality in adult and fetal tissues as well as in placenta, umbilical cord blood and vein, Wharton's jelly and amniotic membrane [6-9]. Recently human umbilical cord blood-derived MSC proved to have the ability to differentiate into endothelial cells in vitro [10].

Amniotic membrane is the innermost layer of placenta and consists of a thin epithelial layer, a thick basement membrane and an avascular stroma. In the amniotic membrane two cell types are present of different embryological origin: amnion epithelial cells derive from embryonic ectoderm and amnion mesenchymal cells from embryonic mesoderm [11,12].

Experimental and clinical studies have demonstrated that amniotic membrane transplantation promotes re-epithelialisation, decreases inflammation and fibrosis [13] and modulates angiogenesis [14]. Several growth factors produced from amniotic membrane are involved in these processes, such as Transforming Growth Factor-β (TGF-β), basic Fibroblast Growth Factor (bFGF) [15], Epidermal Growth Factor (EGF), Transforming Growth Factor-α (TGFα), Keratinocyte Growth Factor, and Hepatocyte Growth Factor [16].

Zhang et al. described the presence of mesenchymal stem cells in human placenta able to differentiate into osteogenic, adipogenic and chondrogenic lineages and able to suppress T-cell proliferation [17]. These results were confirmed by Yen et al., who found that placenta-derived stem cells share Embryonic Stem Cell surface markers such as SSEA-4, TRA-1-61, TRA-1-80 and are also able to undergo neurogenic differentiation. The same authors documented a significantly higher proliferative rate by placenta-derived cells than by their bone marrow counterpart [18].

In 't Anker et al. first showed that amniotic membrane contains a high number of mesenchymal stem cells with bi-potential osteogenic and adipogenic differentiation [19]. Moreover Portsmann-Lanz et al. demonstrated that placental MSCs isolated from the first and third trimester were able to differentiate into chondrogenic, myogenic and neurogenic lineages as well, with major differences among cell types in relation to the different fetal sources (placenta, chorion and amnios) [20]. The opportunity of having a fetal tissue that is usually discarded without any ethical conflict, and the high-yield in stem cell recovery, makes amniotic membrane a truly exciting alternative source, and one that reveals new prospects of increasing the number of clinical applications. Here we characterized the capability of term amniotic membrane-derived cells to differentiate towards adipogenic, chondrogenic, osteogenic, skeletal myogenic lineages and assessed whether these cells may encompass the potential for angiogenic commitment.

In agreement with the recommendation of the International Society for Cellular Therapy, we have named the mesenchymal cells derived from amnion Amniotic Membrane-human Mesenchymal Stromal Cells (AM-hMSCs) [21].

## Results

### Isolation of hMSCs from amniotic membrane

**Figure 1:**
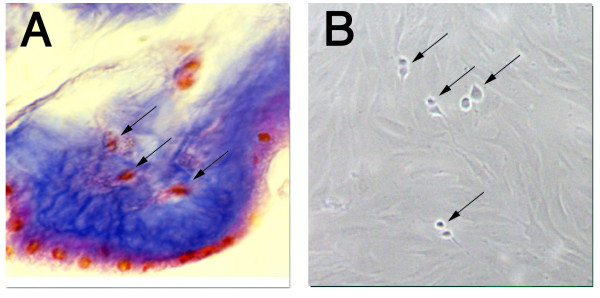
**Human amniotic membrane**. Amniotic membrane sheet as seen by light microscopy. The sample has been stained with Mallory's stain to highlight the connective tissue elements (stained red) as indicated by the arrows (A). Morphology of AM-hMSCs subconfluent at third passage. Magnification ×40. Arrows indicate mitotic figures (B).

Connective tissue elements, seen in the amniotic membrane sheets by light microscopy (Figure 1A), were mechanically and enzymatically released.

After three-four weeks of culture a population of homogeneous mesenchymal cells was isolated from human amniotic membrane and reached confluence. This population was morphologically indistinguishable from the BM-hMSC population and was easily expanded *in vitro *for at least 15 passages without any visible morphological alterations and with a high percentage of mitotic figures (Figure 1B).

**Figure 2:**
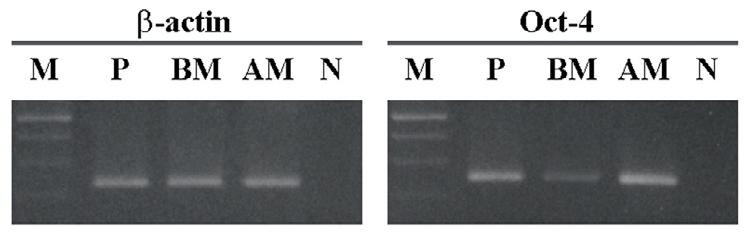
**Oct-4 expression**. RT-PCR analysis of Oct-4 expression in 5^th ^passage AM-hMSCs and BM-hMSCs. Samples are as follows: M: Marker; P: Positive control (HeLa cells); BM: BM-hMSCs; AM: AM-hMSCs; N: Negative control (reagent control). Beta-actin was used as a house-keeping gene. Amplicon lengths: Beta actin 236 bp; Oct-4 249 bp. The data are representative of a set of at least three experiments.

Moreover the presence of the Oct-4 transcript, coding for a relevant regulatory protein involved in the maintaining of stem cell renewal capacity and undifferentiated state in the embryonic stem cells [22], was revealed through RT-PCR analysis in AM-hMSCs and the level of expression was higher than in BM-hMSCs (Figure 2).

**Figure 3:**
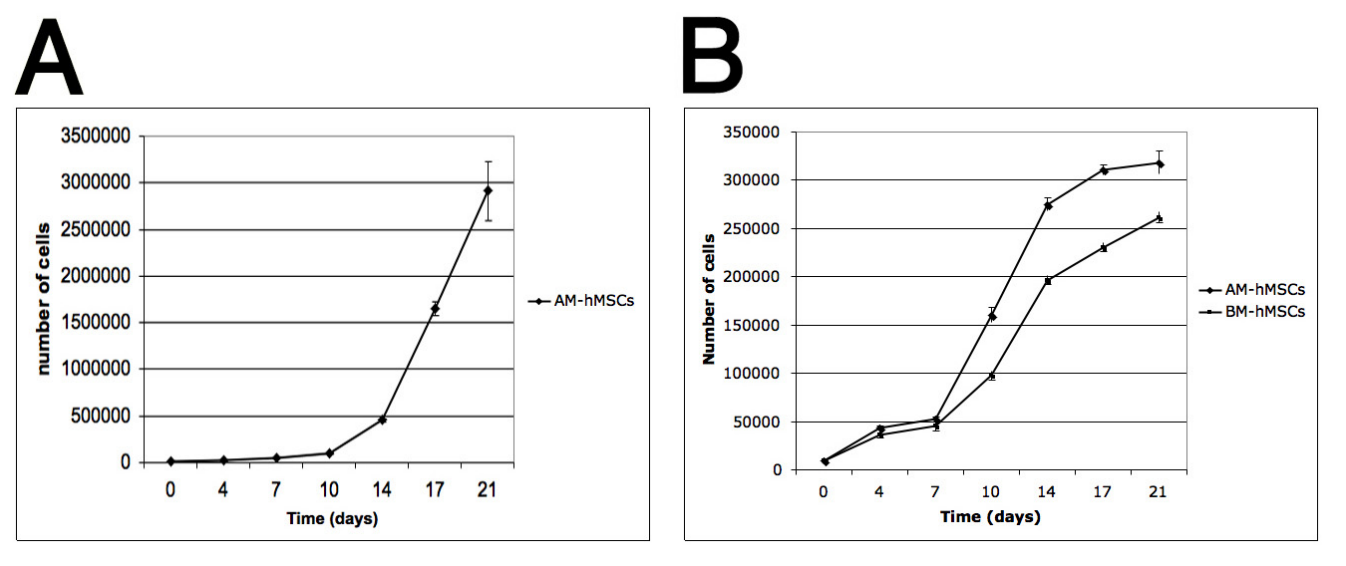
**hMSCs Proliferation assay**. *In vitro *expansion of AM-hMSC. Passage three AM-hMSCs were seeded at an initial concentration of 1000 cells/cm^2 ^(time 0). At day 4, cells were harvested, counted with a hemocytometer and then re-plated at sub-confluent density. The same procedure was repeated at days 7, 10, 14, 17 e 21 (A). Comparison between growth kinetics of amniotic membrane derived cells and bone marrow derived cells. The figure shows the mean numbers of hMSCs obtained by hemocytometer counting on days 0, 4, 7, 10, 14, 17, 21. At t = 0 1000 cells per cm^2 ^were seeded in 6 well plates. Triplicate cultures were harvested for each point. The values represent the mean and SD of three separate experiments (B).

The proliferation capacity of MSCs was studied through 2 different assays: first, AM-hMSCs were maintained in a sub-confluent condition (corresponding to the exponential phase of growth) in order to determine their expansion capacity (Figure 3A). Second, the proliferation rates of AM-hMSCs and BM-hMSCs were compared (Figure 3B). As shown in figure 3A, passage three AM-hMSC expanded approximately 300-fold in 21 days and yielded 2.9 million cells.

Furthermore, at each time point the number of cells in AM-hMSC cultures was significantly higher than in BM-hMSCs (Figure 3B).

### Immunophenotypical characterization

**Figure 4:**
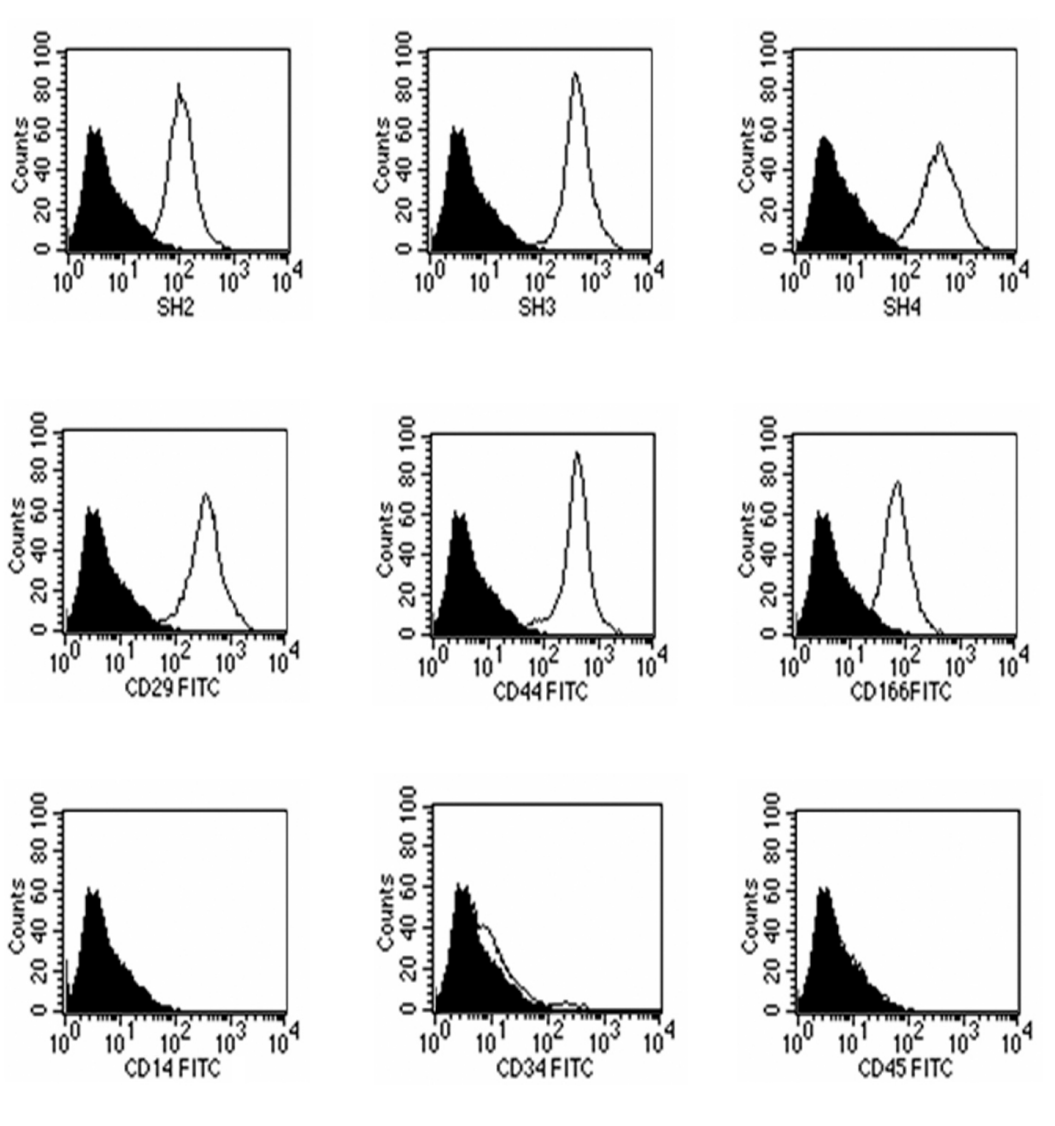
**Immunophenotypical characterization of amniotic membrane derived cells**. Cells at the 5^th ^culture passage were trypsinized, labelled with antibodies against the antigens indicated and analysed by flow cytometry. AM-hMSCs expressed SH2, SH3, SH4, CD29, CD44, CD166, while CD45, CD34, CD14 were negative. A representative example of 5 amniotic membrane samples is shown.

The immunophenotypical profile of AM-hMSCs was consistent with that reported in the literature for BM-hMSCs. Thus characterization by flow cytometry revealed that AM-hMSCs were positive for SH2 (anti-CD105), SH3 and SH4 (anti-CD73), CD29, CD44, CD166, and negative for the hematopoietic markers CD14, CD34, CD45 (Figure 4).

### Differentiation potential

Cells isolated from amniotic membranes were cultured, under specific conditions for targeted commitments, so as to differentiate into multiple cell lineages as described in Methods.

**Figure 5:**
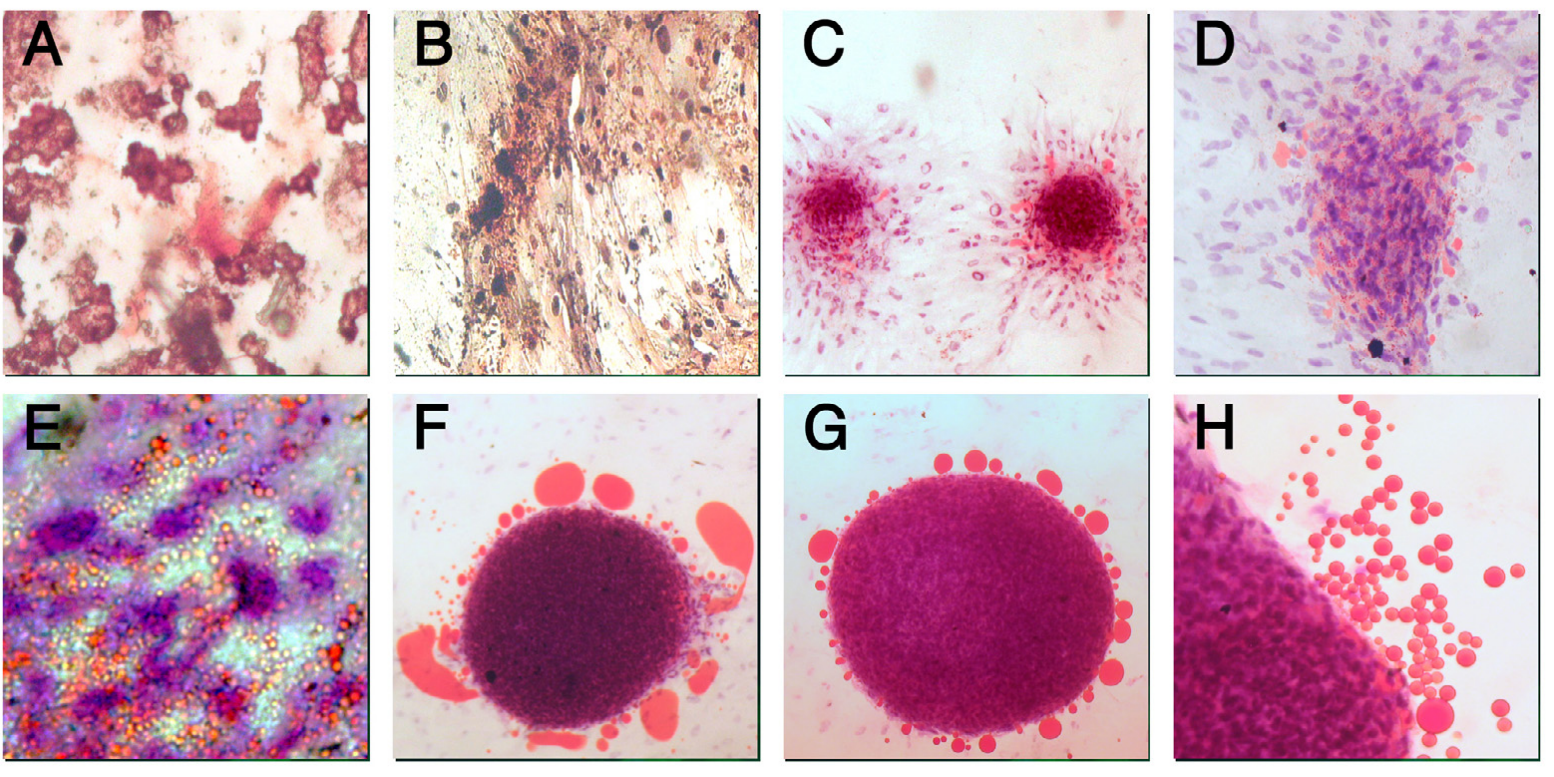
**AM-hMSCs multi-lineage differentiations *in vitro*: chondrogenic (A), osteogenic (B), adipogenic (C-H) commitments**. Chondrogenic differentiation revealed by immunohistochemical stain for collagen II in induced AM-hMSCs. Original magnification ×40 (A). Osteogenic differentiation evidenced by the formation of mineralized matrix as shown by von Kossa staining. Original magnification ×10 (B).Small colonies with lipid secretion during the first week of adipogenic induction as highlighted by Red Oil staining for neutral lipids. Magnification ×4 (C); at higher magnification, multivacuolar adipogenic cells. Magnification ×10 and ×25 respectively (D, E). Big aggregates with intensive and massive lipid secretion at the third week of adipogenic induction. Magnification ×10, ×10 and ×40 respectively (F, G, H).

Chondrogenic differentiation was inferred after 3 weeks' induction by the appearance of abundant extracellular matrix. Such a conclusion was strengthened by immunohistochemical analysis, which showed the presence of human type II collagen (Figure 5A).

Osteogenic differentiation was revealed as early as the first week of induction by morphological changes and, at the end of the induction period, by the formation of mineralized matrix. Cells became flattened and showed calcium deposits as demonstrated by von Kossa staining, suggesting a close similarity with BM-hMSCs (Figure 5B).

No significant differences were found from the *in vitro *differentiation obtained by BM-hMSCs as described in the literature and confirmed in our lab (data not shown).

Adipogenic differentiation showed single adipocytic multivacuolar cells together with small and large colonies, the size increasing with the time of induction. Large-sized aggregates displayed an intensive secretion of large neutral lipid drops which was never observed with BM-hMSC adipogenic differentiation (Figure 5C–H).

No osteogenic, adipogenic or chondrogenic differentiations were seen in the same cells when cultured with the control medium.

**Figure 6:**
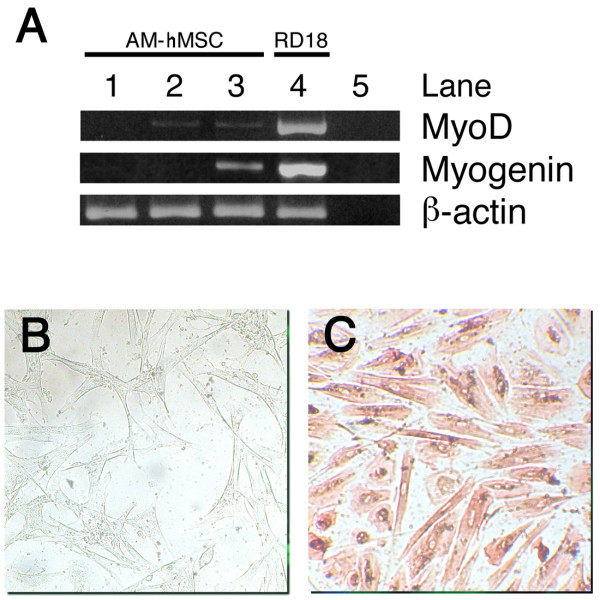
**Skeletal muscle differentiation of AM-hMSCs**. RT-PCR for skeletal muscle transcription factors MyoD and Myogenin. MyoD appears after 1 week of induction while Myogenin is expressed in the second week of induction. Samples are as follows: lane 1: AM-hMSCs cultured in control medium; lane 2: induced AM-hMSCs after 7 days; lane 3: induced AM-hMSCs after 14 days; lane 4: positive control (RD18 cell line); lane 5: reagent control. Beta-actin was used as a house-keeping gene (A). Immunocytochemical staining for Desmin after 3 weeks' induction: uninduced AM-hMSCs (B) and Desmin positive induced cells (C).

Myogenic differentiation was assessed by RT-PCR for the myogenic-specific transcription factors MyoD and Myogenin. As indicated by Muguruma et al. [23], the presence of MyoD was investigated after 7 days' culture, while Myogenin appeared one week later (Figure 6A). The skeletal protein Desmin was detectable after 3 weeks' induction, in line with the fact that lineage-specific cytoskeletal filaments are synthesized during late myogenesis. Immunocytochemical staining was used to highlight desmin-positive cells (Figure 6B,C).

The matrigel assay provided evidence for remarkable features concerning the angiogenic potential.

**Figure 7:**
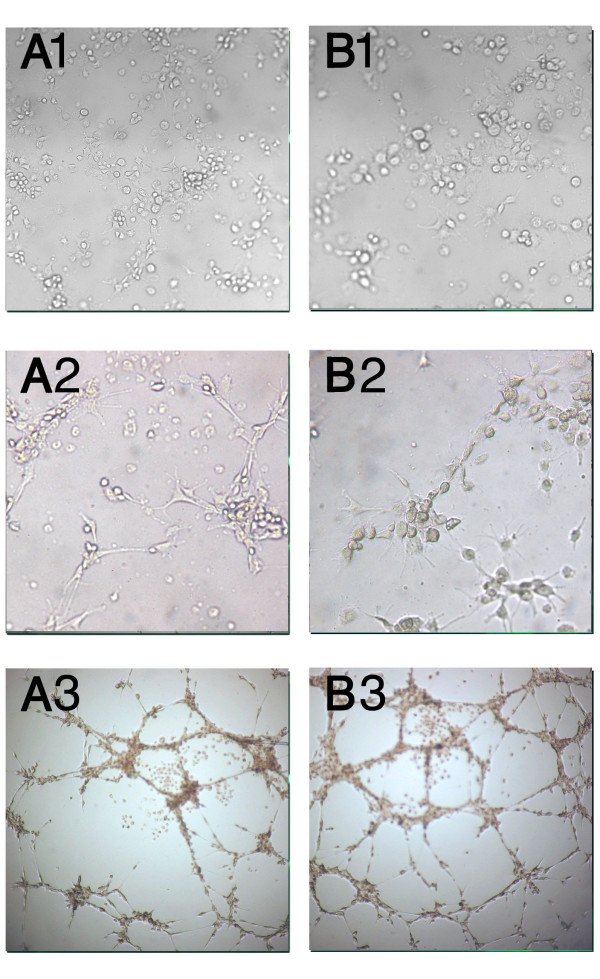
**Angiogenic commitment: light microscopic analysis of AM-hMSCs after incubation on Matrigel**. (A) Spontaneous organization in capillary-like structures on semisolid medium after *2 *(A1), 4 (A2) and 20 (A3) hours of incubation. (B) Increased capillary-like structure formation in AM-hMSCs cultured in angiogenic medium supplemented by VEGF 50 ng/mL after 2 (B1), 4 (B2) and 20 (B3) hours' incubation. Within 4 hours of incubation on semisolid medium, cells preserve a round shape and homogeneous distribution (A1 and B1).

**Figure 8:**
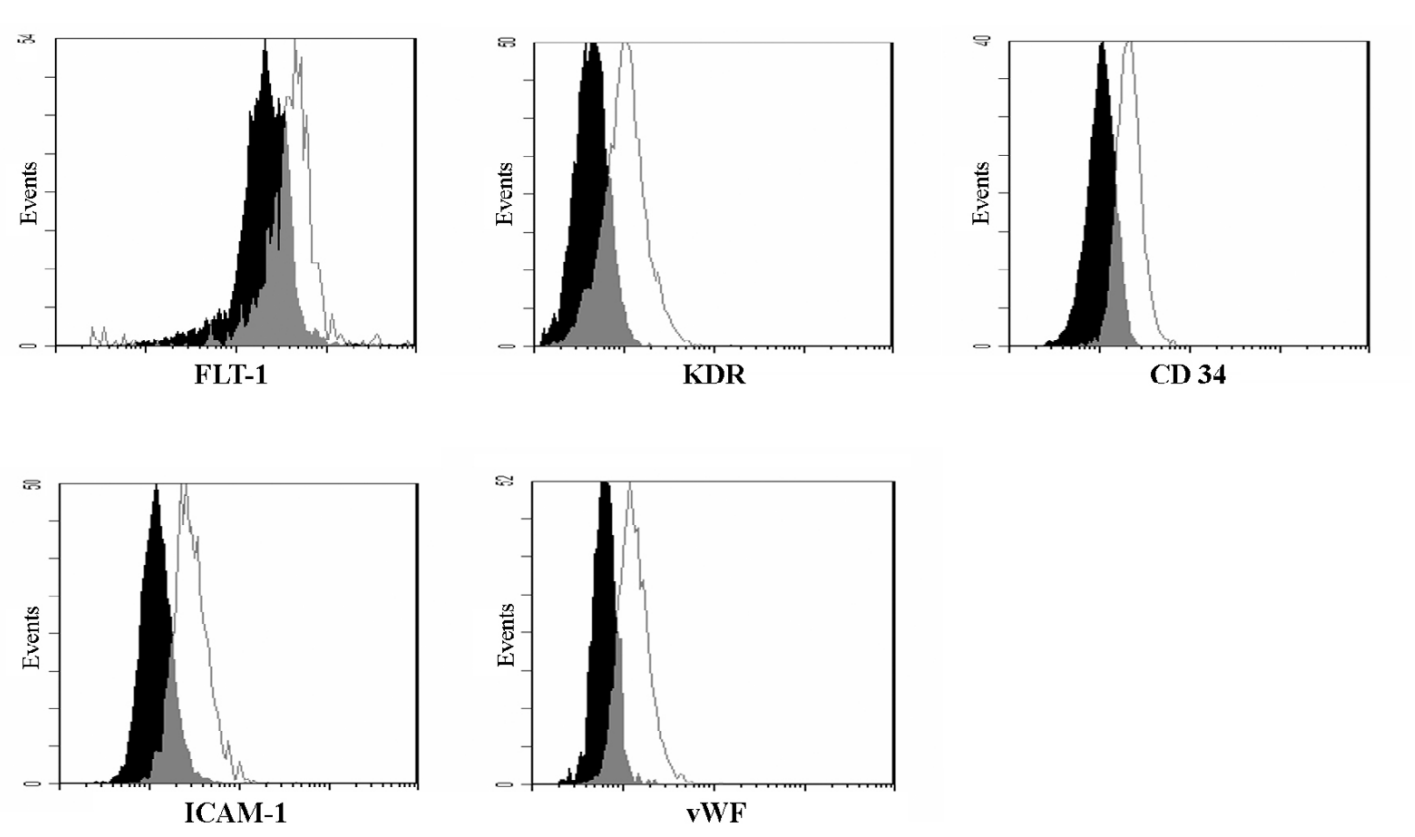
**Endothelial specific markers after AM-hMSCs angiogenic differentiation**. Flow cytometry analysis for FLT-1, KDR, CD34, ICAM-1, vWF expression in AM-hMSCs cultured in absence and in presence of VEGF (50 ng/ml for 7 days). Uninduced cells in black.

**Figure 9:**
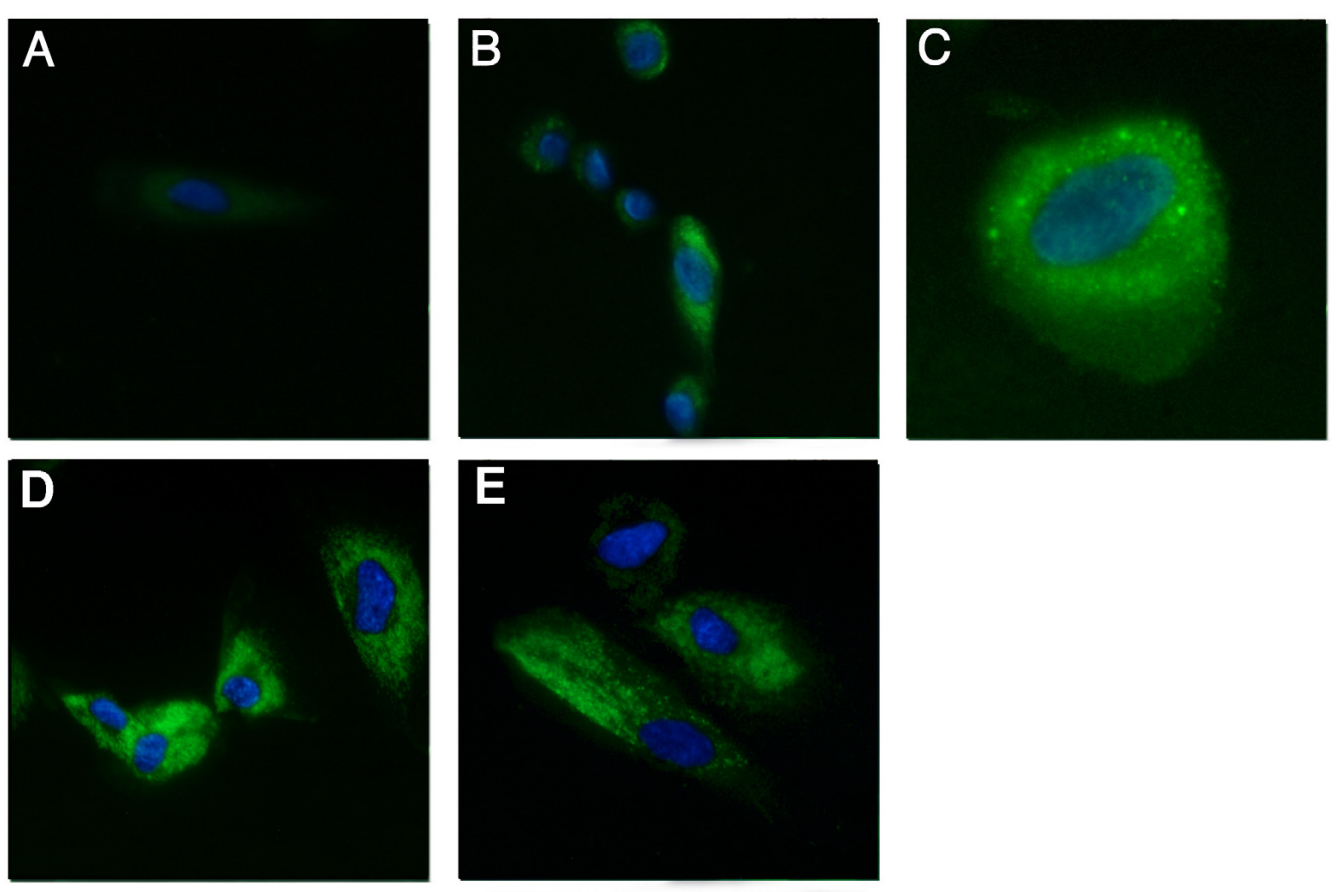
**Immunofluorescence detection of vWF, FLT-1 and KDR expression**. AM-hMSCs cultured in standard medium for 7 days did not show vWF expression (A, Magnification ×40). AM-hMSCs stimulated with medium containing 50 ng/ml of VEGF for 7 days showed vWF expression (B, Magnification ×40); the immunostaining revealed a cytoplasmatic granular positivity at higher magnification (C, Magnification ×100). FLT-1 and KDR expression in induced AM-hMSCs (D, E, Magnification ×40). Nuclei are stained with DAPI (blue).

AM-hMSCs cultured with the specific medium and seeded on top of matrigel (Figures 7A1, B1) showed morphological changes after 4 hours (Figures 7A2, B2 ), while capillary-like structures were evident after 20 hours (Figures 7A3, B3). Moreover, cells maintained viability and tube organization for at least 3 days (data not shown). Unstimulated cells (Figure 7 A3) were able to give rise to capillary-like structures following the same kinetics but with less organizational efficiency than induced cells (Figure 7B3). Analysis of VEGF receptors expression yielded a number of interesting observations. First, VEGF receptors 1 and 2 (FLT-1 and KDR) were both found in AM-hMSCs. Confirming the endothelial differentiation of the cells, flow cytometry analysis revealed an increased expression of FLT-1, KDR, ICAM-1 and the appearance of CD34 when cells had been cultured with VEGF (Figure 8). Akin to these findings, immunofluorescence analysis revealed that cell treatment with VEGF increased the expression levels of both VEGF receptors and was associated with a clear cytoplasmatic granular positivity for von Willebrand Factor (vWF) as compared with untreated cells (Figure 9).

## Discussion

Human mesenchymal stem cells (MSCs) are pluripotent adult stem cells residing within the bone marrow microenvironment. MSCs can differentiate not only into osteoblasts, chondrocytes, adipocytes, neurons, cardiac myocytes, but also into vascular endothelial cells. Despite these appealing properties of bone marrow-derived MSCs, the cell source may become an issue for a broad clinical application of cell therapy, because expansion of autologous bone marrow cells could represent a cumbersome and low-yield approach. Moreover, recent evidence has indicated that age and disease state may affect the collection of sufficient healthy autologous bone marrow for transplantation [24,25]. Thus, the availability of pluripotent cells that can be implanted without manipulation or expansion in an allogenic setting would have obvious biomedical implications and may hold promise for "off the shelf" approaches to cell therapy.

In the present study, we directed our attention towards an alternative source that is easily accessible, high in yield and ethically acceptable: term amniotic membrane. In particular, we succeeded in isolating a multipotent stem cell population that is still of fetal origin and may be superior in proliferation and differentiation potential to cells deriving from adult tissues. Cells were easily isolated through mechanical and enzymatic digestion and showed the fibroblast-like morphology usually observed with mesenchymal stem cells deriving from bone marrow. Furthermore, these cells revealed the characteristic antigen expression pattern of culture-expanded MSCs, since they were positive for the common well-defined hMSC markers [26]. Amniotic membrane is the only source of MSCs without any contamination from non-fibroblastoid cells, unlike what has been described for umbilical cord, cord blood or placenta [18,27,28]. In particular, a variable percentage of hematopoietic and endothelial cells is usually present in cultures of cord blood- or placenta-derived cells, while such contamination is avoided in the case of AM-hMSCs, since amniotic membrane supporting connective tissue is truly devoid of any vasculature.

Moreover, the isolation procedure removes epithelial cells through the first digestion step as demonstrated via morphology analysis by phase-contrast microscopy and in line with the findings of Bilic et al. who used a similar protocol [29].

Amniotic membrane has many additional advantages, since such tissue is usually discarded, yet easily accessible, and allows a very high recovery of cells. Under our experimental conditions, from day 7 of culture onwards, the cellular yield of AM-MSCs obtained from a very small area (about 4 cm^2^) of amniotic membrane was considerably higher than the yield obtained from the same number of bone marrow-derived cells. The number of cells yielded by the primary culture following our procedure (considering 4 cm^2 ^of AM) ranged between 1.3 and 1.5 × 10^6^. Considering the whole area of AM (1300 cm^2^), ideally the cell number obtained could be about 4 × 10^8^, a suitable amount for cell therapy in a human clinical setting [30]. AM-hMSCs expressed the Oct-4 mRNA at higher levels than BM-hMSCs: this is noteworthy, as the Oct-4 transcript is normally found in totipotent embryonic stem cells and the expression of the gene is downregulated during differentiation [22]. The higher level shown by AM-hMSCs could potentially place these cells in a higher position within the stem cell hierarchy compared with MSCs derived from adult tissues.

We confirmed the osteogenic and adipogenic differentiation capacity of AM-hMSCs reported by In't Anker et al. [19]; we also demonstrated that AM-hMSCs are able to differentiate into chondrogenic lineage. Thus amniotic membranes may represent a reservoir of multipotent mesenchymal stem cells every bit as good as bone marrow, while other sources that have been investigated have displayed only a lower differentiation potential. We found, for example, that dental pulp-derived mesenchymal stromal cells failed to differentiate towards the chondrogenic lineage [31].

In adipogenic commitment experiments an unusual growth pattern of adipocytic colonies was observed. During the first 2 weeks of culture, small colonies were found and the accumulation of lipid drops was evident. During the last week of induction, large compact aggregates secreting abundant amounts of neutral lipids became evident. To our knowledge, this intensive secretion has never been described for mesenchymal stem cells deriving from any sources.

These lipid-secreting aggregates could afford an intriguing *in vitro *cell differentiation model for the analysis of adipocytokines. Alterations to such molecules have been found in some diseases involving both the adipogenic and the hemopoietic systems [32].

Again, skeletal muscle differentiation has been obtained with mesenchymal stem cells isolated from lipo-aspirates and umbilical cord blood [33,34].

We demonstrated that AM-hMSCs are able to differentiate towards the skeletal myogenic lineage under physiological culture conditions without the addition of demethylating drugs. These results were comparable to those obtained for human bone marrow-derived multipotent adult progenitor cells by Muguruma et al [23]. These cells could be a candidate for the treatment of muscular dystrophies, for which corticosteroids still remain the only effective treatment [35], due to their easily accessible localization and their high *in vitro *expansion potential.

Myocardial infarction, peripheral arterial occlusive disease with critical ischemia, and stroke are the most important clinical consequences of end-stage occlusive vascular disease for which present-day therapies prove inadequate and treatment remains a palliative.

In this context, the development of a regenerative medicine based on stem cell therapy may hold unprecedented prospects [36-38].

Classical studies by Asahara et al. demonstrated the existence of circulating endothelial progenitor cells (EPCs) within the blood and later their contribution to compensatory vessel growth [39]. More recently human bone marrow-derived and umbilical blood-derived MSCs revealed the ability to differentiate into the endothelial cell lineage after having been conditioned with angiogenic growth factors such as VEGF [5,10]. Therefore it is evident that alternative cell sources for attempting therapeutic neovascularization in adults are available but it should be emphasized that the efficacy of such options remains to be determined in humans.

Here, we provide evidence for the first time that AM-hMSCs display angiogenic potential and may therefore arise as new candidates in cell therapy for vascular diseases. AM-hMSCs spontaneously form capillary-like structures when cultured in semisolid medium (Matrigel system). This behaviour was enhanced by exposure to VEGF, and the endothelial commitment of the cells was demonstrated by the expression of von Willebrand Factor. It should be noted that vWF was only expressed in VEGF-induced cells, so the capillary-like structures formed by uninduced cells are probably due to the hybrid epithelial-mesenchymal ultrastructural phenotype demonstrated in the amniotic membrane [40]. Another interesting finding was that AM-hMSCs expressed CD34 and showed increased expression of FLT-1 and KDR when cultured in the presence of VEGF: such expression, which recapitulates the basic phenotypic profile of circulating Endothelial Progenitor Cells (EPCs), is further evidence that AM-hMSCs have an angiogenic potential; the fact that, under the same culture conditions, AM-hMSCs did not express the CD133 surface molecule helps to distinguish these cells from the bone marrow-derived progenitors [41] (data not shown).

The fact that AM-hMSCs have angiogenic abilities, despite amniotic membrane being an avascular tissue and endothelial cells not being present in the original tissue, raises the issue of whether such an angiogenic potential could be related to the embryonic origin of the hemangioblast. In this respect, it is now evident that in the mouse embryo even the placenta is a site of hemogenic endothelium [42]. An additional worthful finding was the intense expression of ICAM-1 we found following VEGF stimulation; ICAM-1 is a member of the Ig superfamily Ca++ dependent transmembrane proteins, whose increased expression has proved to facilitate leukocyte-mediated angiogenesis [43]. Furthermore, it has recently been found that ICAM-1 is critical in driving cell mobility during angiogenesis [44]; thus it appears that AM-hMSCs not only have the ability to differentiate into endothelial cells but also have some inducible properties which are necessary to regulate angiogenetic processes.

We do not know whether all AM-hMSCs have such angiogenic potential or whether this ability is specific to a subset of endothelial cell progenitors comprised within the AM-hMSC primary isolate. The fact that the amniotic membrane is avascular is against this latter possibility. Moreover, wide-ranging transcriptional profiling with Serial Analysis of Gene Expression (SAGE) performed on single cell-derived colonies of undifferentiated BM-hMSCs showed that a clonal population of hMSCs is molecularly heterogeneous, simultaneously expressing transcripts characteristic of multiple Mesenchymal cell lineages. These observations provided further evidence for the stem-like nature of MSCs [45]. We cannot exclude that these conclusions may also apply to AM-hMSCs. Addressing this issue will require targeted clonogenic studies and is a subject for our future investigations.

## Conclusion

The present observations, focusing on the expansion and differentiation potential of AM-hMSCs, suggest that this population may be a remarkable source of pluripotent cells available for future efforts in cell therapy. BM-hMSCs may not be the elective choice due to the highly invasive donation procedure and the decline in MSC number and differentiation potential with aging. Further studies are required to fully understand the potential of AM-hMSCs as a candidate in cell therapy strategies. Their inducible angiogenic potential may open a new therapeutic approach, based on tissue-engineered vascular grafts, to treating various forms of ischemic vascular diseases.

Therapeutic neo-angiogenesis, may be a strategy to restore tissue damaged because of myocardium infarction, peripheral occlusive vascular diseases, and other different arteriopathies.

In order to develop future treatments based on regenerative medicine, it may be worth noticing that the currently reported yield of AM-hMSCs resulted from a very small area, which is about 1/325 of the total area of amniotic membrane. This result, in addition to the fact that both collagenase and trypsin, which have been used throughout the initial steps of cell isolation, are currently available as GMP-grade enzymes, suggests that a remarkable potential for up-scaling the overall procedure for clinical use could be envisaged [46,47].

## Methods

### hMSC isolation and culture

According to the policy approved by the local Ethical Committee (S.Orsola-Malpighi University Hospital), all tissue samples were obtained after informed consent.

Term placentas from 6 healthy donor mothers obtained from caesarean sections were rapidly transferred to the laboratory, rinsed in Phosphate Buffered Saline (PBS) containing penicillin and streptomycin (200 U/ml penicillin, 200 μg/ml streptomycin) and used immediately.

Amniotic membrane was separated from chorion through blunt dissection. Small fragments were utilized for histological examination. Pieces of amniotic membrane were minced and subjected to 15-minute digestion with 0.25% trypsin-EDTA solution. The supernatant was discarded and the tissue underwent a second digestion with 0.25% trypsin-EDTA solution, 10 U/ml DNAseI and 0.1% collagenase IV solution in DMEM (Dulbecco's modified Eagle's medium) (all purchased from Sigma-Aldrich Co., St. Louis, USA). The fragments were pipetted vigorously up and down for 5 minutes, avoiding foam; larger pieces of tissue were allowed to settle under gravity for 5 minutes at 37°C. Each supernatant was transferred to a fresh tube, neutralized with Fetal Bovine Serum (FBS, Biochrom, Berlin, Germany), then spun at 1500 rpm for 10 minutes. Each pellet was re-suspended in 5 ml of culture medium containing: DMEM, 20% FBS, penicillin 100 U/ml and streptomycin 100 μg/ml. Cells were seeded in 25 cm^2 ^flasks and the hMSC cultures grew at 37°C in 5% CO_2_. Non-adherent cells were removed after 1 week and the medium (with 10 % of FBS) was subsequently changed every four days. When the culture reached 90% confluence, cells were recovered using 0.25% Trypsin-EDTA for flow cytometric analysis, expansion or other studies.

hMSCs isolated from bone marrow (BM-hMSCs) were collected from 5 healthy adult volunteers (mean age 45 years; male 3, female 2) in accordance with the protocol previously described [31].

### Flow cytometric analysis

For flow cytometric analysis, the fibroblast-like cells obtained from amniotic membrane were harvested at the same point of culture by treatment with 0.25% trypsin-EDTA and incubated with 1 μg/10^6 ^cells FITC-conjugated antibodies for 40 minutes at 4°C in the dark. The antibodies used were: SH2, SH3 and SH4 kindly provided by Dr. Mark Pittenger (Osiris Therapeutics, Baltimore, MD, USA); anti-CD29, anti-CD44 and anti-CD166 (Ancell, Bayport, MN, USA); anti-CD14, anti-CD34 and anti-CD45 (Becton Dickinson, San Jose, CA, USA). After washing, cells were analyzed on a flow cytometer (FACSCalibur, Becton Dickinson, San Jose, CA, USA) collecting 10,000 events and the data analyzed by Cell Quest Software (Becton Dickinson, San Jose, CA, USA).

### Molecular analysis

Total RNA was extracted from 5 × 10^5 ^cells (5^th ^passage BM-hMSCs and AM-hMSCs, HeLa cells as a positive control) using TRIzol^® ^Reagent according to the manufacturer's instructions (Invitrogen, Carlsbad, CA, USA) [48]. Reverse transcription reactions were performed in a 20 μl volume with 2 μg of total RNA using Cloned AMV First-Strand cDNA Synthesis Kit^® ^(Invitrogen) following the manufacturer's protocol. The expression of the following mRNAs was investigated: Oct-4, Beta actin (control). Primers used in the RT-PCRs were: 1) Oct-4: annealing temperature: 60°C, amplicon length 249 bp, sense 5'-CGT GAA GCT GGA GAA GGA GAA GCT G-3', antisense 5'-CAA GGG CCG CAG CTT ACA CAT GTT C-3'; 2) Beta actin: annealing temperature: 55°C, amplicon length 236 bp, sense 5'-GGA CTT CGA GCA AGA GAT GG-3', antisense 5'-AGC ACT GTG TTG GCG TAC AG-3'

The PCR products were visualized on a 1,5% agarose gel stained by ethidium bromide.

### Proliferation assay

Passage three AM-hMSCs were seeded at an initial concentration of 1000 cells/cm^2 ^in a 6-well plate. At day 4, cells were harvested with 0.25% Trypsin-EDTA solution for 3 minutes at 37°C, counted with a hemocytometer and then re-plated at sub-confluent density. The same procedure was repeated at days 7, 10, 14, 17 e 21.

hMSCs derived from amniotic membrane and from bone marrow were cultured at 1000 cells/cm^2 ^in duplicate in a 6-well plate. Cells were detached by treatment with Trypsin-EDTA and counted with a hemocytometer at days 4, 7, 10, 14, 17 e 21 by Trypan blue exclusion (Sigma-Aldrich Co., St. Louis). Both of these experiments were performed 3 times for each point described.

### *In vitro *differentiation studies

At the third culture passage, AM-hMSCs were induced to differentiate into five different types of cell: adipocytes, osteoblasts, chondroblasts, skeletal muscle cells and endothelial cells. Differentiation studies were performed in parallel with BM-hMSCs. (data not shown).

*In vitro *adipogenic, osteogenic and chondrogenic differentiations were induced following the protocols previously described [31].

#### Chondrogenic differentiation of AM-hMSCs

To induce chondrogenic differentiation, aliquots of 5 × 10^5 ^cells were pelleted in polypropylene conical tubes in 0.5 ml of DMEM containing 6.25 μg/ml insulin, 6.25 μg/ml transferrin, 6.25 μg/ml selenous acid, 5.33 μg/ml linolenic acid, 1.25 mg/ml BSA, 0.35 mM proline, 1 mM sodium pyruvate, 10^-7^M dexamethasone, 0.1 mM L-ascorbic acid-2-phosphate (all Sigma), supplemented with 10 ng/ml Transforming Growth Factor-β 3 (TGF-β 3 R&D Systems, Minneapolis, MN, USA). This medium was replaced every 3–4 days for 3–4 weeks. Pellets were formalin-fixed, embedded in paraffin, examined morphologically and immunostained for Type II collagen, using Vectastain elite ABC kit (Vector Laboratories, Burlingame, CA, USA) (Chemicon Int, Tamecula, CA, USA).

#### Osteogenic differentiation of AM-hMSCs

To induce osteogenic differentiation, 3 × 10^3 ^cells/cm^2 ^were plated in chamber slides (NUNC) in DMEM (Sigma) supplemented with 10% FBS, 10 mM β-glycerophosphate, 0.2 mM ascorbic acid, and 10^-8 ^M dexamethasone (Sigma), and cultured for 3–4 weeks, replacing the medium every 2–3 days. To demonstrate osteogenic differentiation, the cultures were fixed and von Kossa stained.

#### Adipogenic differentiation of AM-hMSCs

To induce adipocyte differentiation, 10 × 10^3 ^cells/cm^2 ^were cultured in DMEM supplemented with 10% FBS, 0.5 mM isobutyl-methyl xanthine (IBMX), 200 μM indomethacin, 10^-6^M dexamethasone and 10 μg/ml insulin (all Sigma) in chamber slides (NUNC, Naperville, IL, USA). The cells were cultured, replacing the medium every 2–3 days. After 2–3 weeks of culture the cells contained neutral lipids in fat vacuoles; they were fixed in 10% formalin and stained with fresh oil red-O solution (Sigma).

#### Myogenic differentiation of AM-hMSCs

*In vitro *myogenic differentiation was induced according to the protocol used for human bone marrow-derived multipotent adult progenitor cells [23]. 5 × 10^3 ^cells/cm^2 ^were plated in chamber slides or 25 cm^2 ^flasks in DMEM supplemented with: 5% of FBS, 40% MCDB-201, ITS-LA*BSA 100X, 10^-8^M dexamethasone, 10^-4^M ascorbic-acid-2-phosphate, 10 ng/ml bFGF (R&D Systems), 10 ng/ml Vascular Endothelial Growth Factor (VEGF: Sigma), 10 ng/ml Insulin like Growth Factor-1 (R&D Systems). This medium was replaced every 3–4 days for 3 weeks. At day 7 and day 14 total RNA was extracted for RT-PCR in order to investigate the expression of MyoD and myogenin respectively. Total cellular RNA was extracted by RNeasy Mini kit (QUIAGEN, Milan, Italy). First strand cDNA was synthesized with 1 μg of total RNA in a reverse buffer containing 100 pmol of oligo dT, dNTPs 0.25 mM, DTT 10 mM, 20 U of Rnase Inhibitor and 50 U of M-MuLV RT. The mixture was incubated for 1 hour at 42°C, heated for 5 minutes at 94°C and stored at -20°C until used. PCR amplifications were carried out in a thermal cycler with cDNA derived from 200 ng of total RNA, reaction buffer 10×, MgCl_2 _2 mM, dNTP 0.2 mM, primers 0.2 mM and 1.25 U of Taq DNA polymerase. Primers were designed and synthesized for Myo-D (sense 5'-AAG CGC CAT CTC TTG AGG TA-3'; antisense 5'-GCG CCT TTA TTT TGA TCA CC-3') (Invitrogen, Milan, Italy) and Myogenin (sense 5'-AGG CTC AAG AAG GTG AAT GAG G-3'; antisense 5'-AGG TTG TGG GCA TCT GTA GGG T-3'). Myogenin primers were kindly provided by Prof Carla De Giovanni (section of Cancer Research, Department of Experimental Pathology, University of Bologna, Italy) [49]. Each cycle included a denaturation step (95°C for 1 min), an annealing step (60°C for 1 min) and an extension step (72°C for 1 min). The PCR products were visualized on a 2% agarose gel stained by ethidium bromide.

A cell line deriving from rhabdomyosarcomas (RD18) was used as a positive control for skeletal myogenic markers.

After 3 weeks' induction AM-hMSCs were fixed with Aceton:Methanol = 7:3 and analysed by immunocytochemistry assay in order to investigate the presence of desmin protein. In particular a Vectastain elite ABC kit (Vector Laboratories) was used with primary anti-Desmin antibody (Sigma).

#### Angiogenic differentiation

Confluent cells were cultured in DMEM with 2%FBS and 50 ng/ml VEGF for 7 days, changing the medium every 2 days [4]. AM-hMSCs cultured in DMEM with 10% FBS for the whole induction period were considered the negative control.

Analysis of capillary formation was performed using Matrigel (Becton Dickinson and Co, Franklin Lakes, New Jersey, USA). 50 μl of gel matrix solution was applied to each well on a 96-well plate and incubated for 1 hour at 37°C. 1 × 10^4 ^cells were suspended in 50 μl of DMEM, plated onto the gel matrix and incubated at 37°C. Capillary-like structures were observed by optical microscopy after 2, 4 and 20 hours and at regular intervals during the following 3 days.

In order to evaluate the FLT-1, KDR and von Willebrand Factor (vWF) expression, AM-hMSCs cultured as above described were harvested, counted and seeded on cover slips in 6-well plates at a concentration of 5000 cells/cm^2^. Cells were allowed to adhere overnight, then immunofluorescence stain was performed as briefly described. Cells were washed with PBS and fixed with 2% paraformaldehyde for 4 minutes at room temperature. For intracytoplasmatic protein staining (vWF), 1% triton X-100 was added during the fixation. Aspecific antibody binding sites were blocked by incubating with 1% bovine serum albumin for 1 hour at 37°C. After blocking cells were labelled for 45 minutes at 37°C in the presence of a mouse monoclonal antibody (dilution of 1:50) directed against vWF (DakoCytomation, Denmark), a goat polyclonal antibody (diluition of 1:50) direct against FLT-1 (Santa Cruz Biotechnology-USA), a mouse monoclonal antibody (diluition of 1:50) directed against KDR (SigmaAldrich, Italy). Excess primary antibody was removed by six washes with PBS and the cells were stained at 37°C for 45 minutes with a rabbit fluorescein-conjugated anti mouse IgG (DakoCytomation, Denmark) and with a donkey fluorescein-conjugated anti-goat IgG (Santa Cruz Biotechnology, USA). Nuclei were labelled by adding a drop of Pro Gold Antifade with DAPI (InvitrogenMolecular Probes, Italy).

Cells adhering onto cover slips were observed and photographed under a microscope (Axiovert 40 Zeiss, Germany) with UV lamp.

The AM-hMSCs from the same angiogenic experiment were analyzed, in parallel assays, by flow cytometry using the following antibodies according to the manufactorers' suggestions: FLT-1 (Santa Cruz Biotechnology-USA), KDR (RD System, Minneapolis, USA), CD34 (Beckman-Coulter, Miami, USA), ICAM-1 (Beckman-Coulter, Miami, USA) and vWF (DakoCytomation, Denmark). In order to reveal vWF, the cells were permeabilized with the Intrapep Kit (Beckman-Coulter), incubated with vWF MoAb and subsequently incubated with secondary anti-mouse IgG FITC (DakoCytomation-Denmark).

Data were acquired using a Cytomics FC500 Flow Cytometer equipped with two sources (Beckman-Coulter). Results were analyzed using the CXP Software (Beckman-Coulter).

## Authors' contributions

FA and VF carried out the MSC isolation and differentiation, CM performed RT-PCR assays, MA and PLT designed and performed the cytometric analysis, LB, AG, CV participated in designing the study, MF provided placenta samples, GL, GP and LF carried out angiogenic differentiation and SC, CC and FB myogenic differentiation, GPB participated in the design and coordination. All authors read and approved the final manuscript.
